# Immunohistochemical Approach to Study Cylindrospermopsin Distribution in Tilapia (*Oreochromis niloticus*) under Different Exposure Conditions

**DOI:** 10.3390/toxins6010283

**Published:** 2014-01-08

**Authors:** Remedios Guzmán-Guillén, Daniel Gutiérrez-Praena, María de los Ángeles Risalde, Rosario Moyano, Ana Isabel Prieto, Silvia Pichardo, Ángeles Jos, Vitor Vasconcelos, Ana María Cameán

**Affiliations:** 1 Area of Toxicology, Faculty of Pharmacy, University of Seville, Profesor García González 2, Seville 41012, Spain; E-Mails: rguzman1@us.es (R.G.-G.); anaprieto@us.es (A.I. P.); spichardo@us.es (S. P.); angelesjos@us.es (A. J.); camean@us.es (A.M. C.); 2 Department of Anatomy and Comparative Pathology and Anatomy, University of Córdoba, Campus de Rabanales, Carretera Madrid-Cádiz s/n, Córdoba 14071, Spain; E-Mail: risalde10@hotmail.com; 3 Department of Pharmacology, Toxicology and Legal and Forensic Medicine, University of Córdoba, Campus de Rabanales, Carretera Madrid-Cádiz s/n, Córdoba 14071, Spain; E-Mail: ft1mosam@uco.es; 4 Laboratory of Ecotoxicology, Genomics and Evolution, Interdisciplinary Center of Marine and Environmental Research—CIIMAR/CIMAR, University of Porto, Rua dos Bragas 289, Porto 4050-123, Portugal; E-Mail: vmvascon@fc.up.pt; 5 Department of Biology, Faculty of Sciences, Porto University, Porto 4069-007, Portugal

**Keywords:** Cylindrospermopsin, *Aphanizomenon ovalisporum*, *Oreochromis niloticus*, immunohistochemistry, distribution, cyanobacteria, cyanotoxin

## Abstract

Cylindrospermopsin (CYN) is a cytotoxic cyanotoxin produced by several species of freshwater cyanobacteria (*i.e.*, *Aphanizomenon ovalisporum*). CYN is a tricyclic alkaloid combined with a guanidine moiety. It is well known that CYN inhibits both protein and glutathione synthesis, and also induces genotoxicity and the alteration of different oxidative stress biomarkers. Although the liver and kidney appear to be the main target organs for this toxin based on previous studies, CYN also affects other organs. In the present study, we studied the distribution of CYN in fish (*Oreochromis niloticus*) under two different exposure scenarios using immunohistochemical (IHC) techniques. In the first method, fish were exposed acutely by intraperitoneal injection or by gavage to 200 µg pure CYN/Kg body weight (bw), and euthanized after 24 h or five days of exposure. In the second method, fish were exposed by immersion to lyophilized *A. ovalisporum* CYN-producing cells using two concentration levels (10 or 100 µg/L) for two different exposure times (7 or 14 days). The IHC was carried out in liver, kidney, intestine, and gills of fish. Results demonstrated a similar pattern of CYN distribution in both experimental methods. The organ that presented the most immunopositive results was the liver, followed by the kidney, intestine, and gills. Moreover, the immunolabeling signal intensified with increasing time in both assays, confirming the delayed toxicity of CYN, and also with the increment of the dose, as it is shown in the sub-chronic assay. Thus, IHC is shown to be a valuable technique to study CYN distribution in these organisms.

## 1. Introduction

Cyanobacteria, also known as blue-green algae, have the ability to form dense blooms in certain situations, decreasing the water quality. Some bloom-forming species of cyanobacteria are able to produce harmful secondary metabolites called cyanotoxins, causing undesirable effects with implications in human and animal health via drinking and recreational waters or consumption of contaminated food through the food web [1].

Cylindrospermopsin (CYN) is one cyanotoxin present in freshwater bodies around the world and has caused environmental concern. CYN is produced by several species of cyanobacteria, among them *Aphanizomenon ovalisporum* [2]. Structurally, it is an alkaloid consisting of a tricyclic guanidine moiety combined with hydroxymethyluracil [3]. Due to its physicochemical properties, CYN is highly water-soluble [4] and very stable under different environmental conditions [5].

Funari and Testai [6] established that the main targets of CYN are the liver and kidney, but it is considered a cytotoxin since other organs may also be affected after an exposure to the toxin [7,8,9]. Toxicity of CYN is mediated by the inhibition of protein [7] and glutathione synthesis [10], genotoxicity mediated by DNA fragmentation [11], and also by the induction of oxidative stress [12]. Cylindrospermopsin may also inhibit progesterone production, recognizing that the toxin has some potential for endocrine disruption [13].

The possibility of CYN accumulation in aquatic organisms through the trophic web is a serious concern, since many aquatic organisms are for human consumption. Field and laboratory studies on presence and accumulation of CYN in these aquatic organisms are scarce [14]. White *et al*. [15] studied the accumulation of CYN in the aquatic snail *Melanoides tuberculata* exposed to an extract from a CYN-producer *Cylindrospermopsis raciborskii* strain and also to a living culture of these cyanobacteria. Results showed that CYN from live cells presented higher tissue accumulation. In addition, Berry and Lind [16] evaluated the presence of CYN in the snail *Pomacea patula catemacensis*. Regarding bivalves, accumulation up to 560 μg CYN/kg fresh weight was found in the mussel *Alathyria pertexta pertexta*, collected from a reservoir containing <0.8 μg CYN/L [17]. Saker *et al*. [18] also demonstrated CYN accumulation in different tissues from the swan mussel, *Anodonta cygnea*, exposed to 14–90 μg CYN/L for 16 days. Saker and Eaglesham [19] reported the accumulation of CYN in the hepatopancreas and muscle of the redclaw crayfish *Cherax quadricarinatus* collected in an aquaculture pond with a severe bloom of *C.* *raciborskii*. They also demonstrated that this accumulation occurred to a lesser extent in *in vivo* studies in the laboratory. White *et al*. [20] studied the accumulation of CYN in tadpoles of *Bufo marinus* exposed to an extract of a CYN-producer *C. raciborskii* culture or to a culture of *C. raciborskii*, indicating the bioaccumulation of CYN, mainly via grazing. Finally, fish are able to accumulate CYN by direct feeding, by the uptake of the dissolved toxin through gills or skin, or by exposure through the food web. For the first time, Messineo *et al*. [21] found a moderate CYN accumulation in wild trout tissues. More recently, Berry *et al*. [22] showed that CYN accumulation in several fish species mainly occurred in the muscle. Thus, the general order for organisms’ bioaccumulation ability has been established by Kinnear [23] as follows: gastropods > bivalves > crustaceans > amphibians > fish.

When referring to CYN toxicity, there are different issues to consider, such as the variability between animal species and even between the individuals of the same species [23] because of their idiosyncrasies. In addition, the different toxicity induced by pure CYN or CYN cyanobacterial extracts, is another matter to take into account. Results show that the toxicity induced by the toxin from the extracts is higher than that produced by the pure CYN. This could be explained by the presence of other compounds in the cyanobacterial extract [8,24,25]. Hawkins *et al*. [26] showed that pure CYN mainly affected the liver, while crude extracts of *C.* *raciborskii* administered by intraperitoneal (i.p.) injection or oral route to mice also induced pathological symptoms in kidney, lungs, stomach, spleen, thymus, heart, and the vascular and lymphatic systems. Berry *et al*. [27] concluded that direct immersion of zebrafish embryos in extracts from several isolates of *C.* *raciborskii* and *A.* *ovalisporum* resulted in high toxicity, suggesting that these extracts could contain some toxic metabolites other than CYN. Recently, our group has demonstrated that acute exposure to pure CYN by oral route (gavage) and i.p. injection induced a dose- and a time-dependent oxidative stress and histopathological effects in different organs of tilapia (*Oreochromis* *niloticus*) [28,29,30]. It has also been proved that CYN from a cyanobacterial extract produced severe injuries in comparison to the pure toxin [31]. As Kinnear [23] described, the reverse order regarding the bioaccumulation has been established for the susceptibility of the organisms to CYN.

In order to assess CYN distribution in tissues, immunohistochemistry (IHC) is an interesting technique based on the principle of antibodies binding specifically to antigens. IHC has been useful for detecting tissue distribution of Microcystin-LR (MC-LR) in mice [32,33], rainbow trout [34] and in various organs of the gastropod *Lymnaea stagnalis* [35]. Furthermore, it has been employed in the detection of different viruses in cell cultures [36] and tissues [37], as well as in the diagnosis of illnesses [38]. In any case, these studies only refer to acute toxicity but, taking into account that fish are naturally exposed to cyanobacterial blooms for sub-chronic and chronic periods, it would be of interest to compare the effects of acute and sub-chronic exposures to CYN by different routes. 

Therefore, the aim of this study was to examine and compare the tissue distribution of CYN in various organs from tilapia (*O. niloticus*) by IHC. Two different exposure scenarios were used: (a) acute exposure by oral route (gavage) and i.p. injection to a single dose of 200 µg/Kg bw of pure CYN for 24 h and five days; (b) sub-chronic exposure to different concentrations of CYN (10 and 100 µg CYN/L) contained in a lyophilized *A. ovalisporum* culture obtained from a natural cyanobacterial bloom by immersion for 7 and 14 days.

## 2. Results and Discussion

### 2.1. Results

#### 2.1.1. Acute Dose Assay

In the positive control tissue samples, CYN-specific labeling appeared as evenly distributed dark red granules or as diffuse homogeneous staining distributed in the cytoplasm of the cells. The distribution of CYN, mainly associated with the organs studied, is summarized in Table 1.

**Table 1:** Distribution of immunolabeled cells for CYN in different organs from tilapia (*Oreochromis niloticus*) exposed to a single dose of 200 µg/Kg bw of pure CYN and euthanized 24 h or five days after the exposure. Results are expressed as number of immunolabeled cells per area of 0.2 mm^2^: - absent, + scarce (0–10), ++ moderate (10–50), +++ intense (>50). **OC:** Control fish treated with a saline solution (0.9% NaCl) by the oral route; **IPC:** Control fish treated with a saline solution (0.9% NaCl) by the i.p. route; **OI24h:** Fish exposed to a single dose of 200 µg/Kg bw of pure CYN by the oral route and euthanized after 24 h; **IPI24h:** Fish exposed to a single dose of 200 µg/Kg bw of pure CYN by the i.p. route and euthanized after 24 h; **OI5d:** Fish exposed to a single dose of 200 µg/Kg bw of pure CYN by the oral route and euthanized after five days; **IPI5d:** Fish exposed to a single dose of 200 µg/Kg bw of pure CYN by the i.p. route and euthanized after five days.

	OC	IPC	OI24h	IPI24h	OI5d	IPI5d
**Liver**						
Hepatocytes	-	-	-	+	+	++
Pancreatic acini	-	-	+	++	+	+
Erythrocytes	-	-	-	-	+	++
**Kidney**						
Tubules	-	-	-	-	+	+
Glomeruli	-	-	-	-	+	++
Erythrocytes	-	-	-	-	-	+++
**Intestine**						
Epithelium	-	-	-	-	-	++
**Gills**						
Secondary lamellae	-	-	-	-	++	+
Erythrocytes	-	-	-	-	-	+

**Figure 1:**
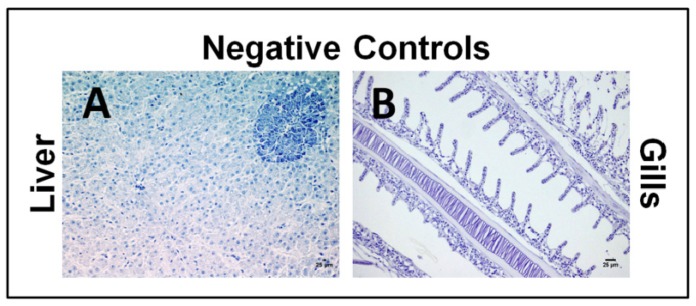
Photomicrographs of the immunohistochemistry of liver (**A**) and gills (**B**) from positive tissue sections of tilapia exposed to CYN where the primary antibody was replaced by PBS or by rabbit non-immune serum, respectively. Bars: 25 µm. No labeling and residual endogenous peroxidase activity were observed in any of these technical negative controls.

**Figure 2:**
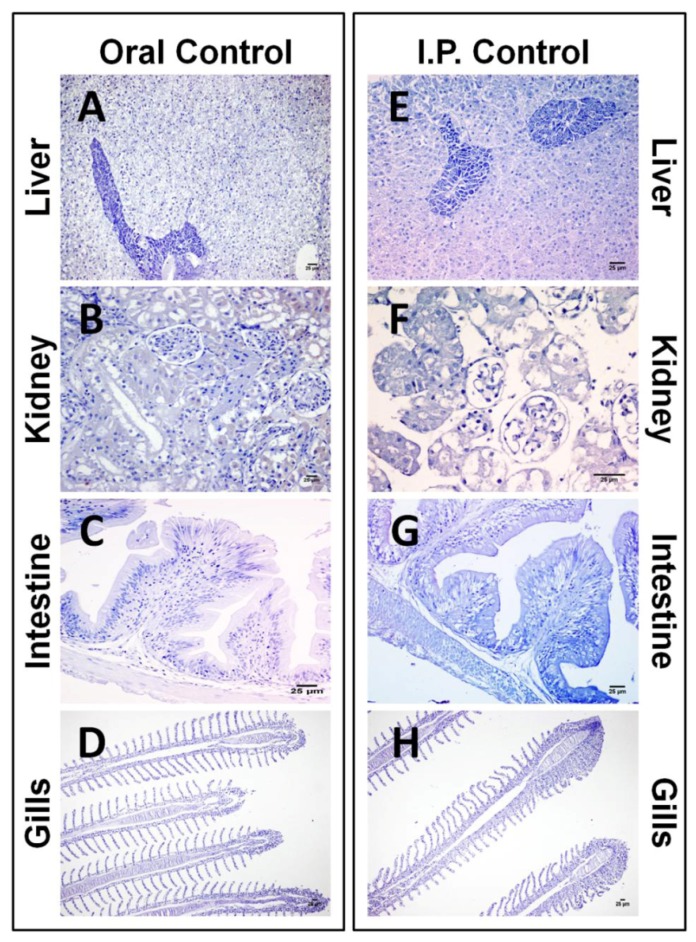
Photomicrographs of the immunohistochemistry of CYN in liver (**A**,**E**), kidney (**B**,**F**), intestine (**C**,**G**), and gills (**D**,**H**) from tilapia used as control in the acute assay. Bars: 25 µm. No labeling was observed in any of the control tissues.

There was no CYN-specific labeling in any control samples when rabbit anti-CYN serum was replaced by rabbit non-immune serum or PBS in the immunohistochemical study (Figure 1). CYN antigen was not detected in any of the tissue sections analyzed from fish not exposed to the toxin (Figure 2).

**Figure 3:**
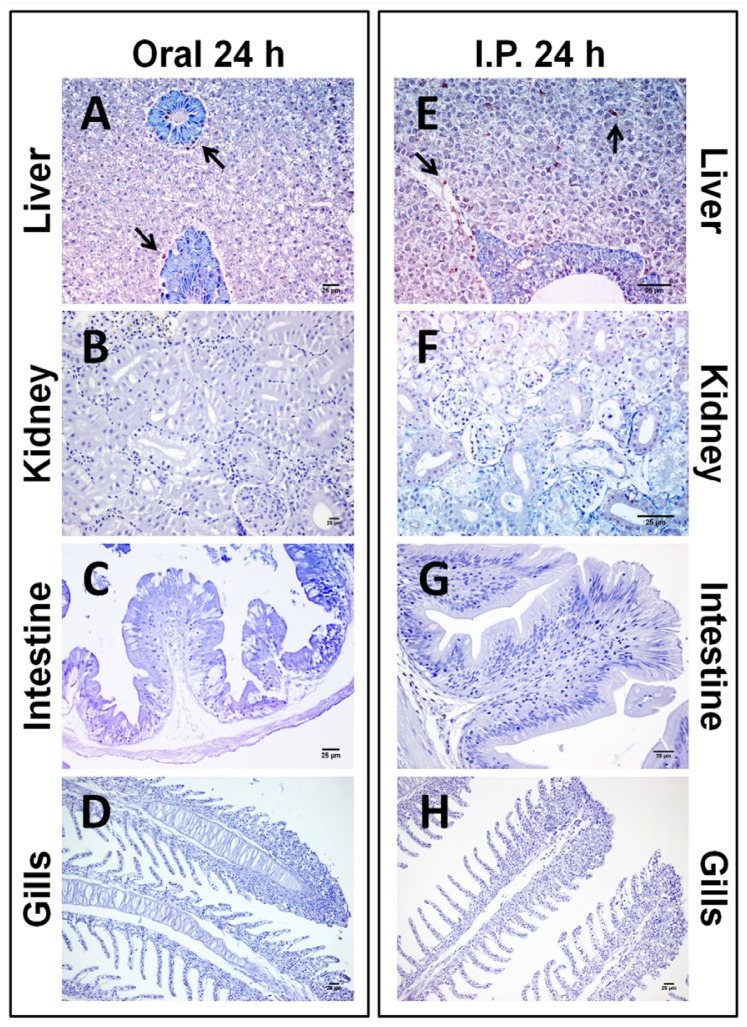
Photomicrographs of immunohistochemistry of CYN in liver (**A**,**E**), kidney (**B**,**F**), intestine (**C**,**G**), and gills (**D**,**H**) from tilapia exposed orally (**A**–**D**) or intraperitoneally (**E–H**) to 200 µg/kg bw of pure CYN and euthanized after 24 h. Bars: 25 µm. (**A**) Liver with scarce CYN-immunopositive staining; (**B**) Normal renal parenchyma without CYN-positive staining; (**C**) No immunostaining in the intestine; (**D**) Normal gills parenchyma after the exposure; (**E**) CYN-positive staining of the cytoplasm of the hepatocytes from tilapia exposed by i.p. injection; (**F**) Renal parenchyma with an intense immunostaining; (**G**) Absence of staining in the epithelium of the intestine; (**H**) Isolated CYN-immunopositivity in gills from fish intraperitoneally exposed. Arrows indicate CYN-immunopositive cells.

**Figure 4:**
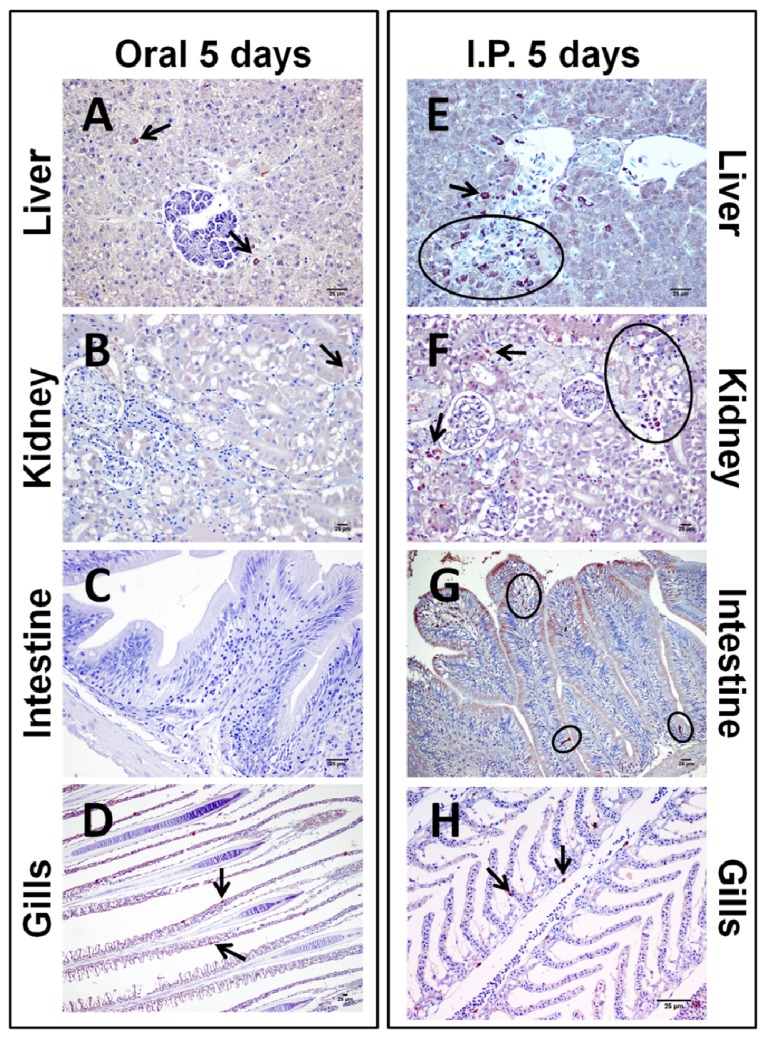
Photomicrographs of immunohistochemistry of CYN in liver (**A**,**E**), kidney (**B**,**F**), intestine (**C**,**G**), and gills (**D**,**H**) from tilapia exposed orally (**A–D**) or intraperitoneally (**E–H**) to 200 µg/kg bw of pure CYN and euthanized after five days. Bars: 25 µm. (A) Hepatocytes from liver with a light CYN-immunostaining; (**B**) Positive staining of the renal parenchyma mainly localized in proximal and convoluted tubules; (**C**) No immunostaining in the intestine; (**D**) Normal gills parenchyma after the exposure; (**E**) Moderate CYN-positive staining the hepatocytes; (**F**) Intense immunostaining in cells from the kidney principally localized in glomeruli and erythrocytes; (**G**) Slight immunostaining in the intestine, mainly localized in the epithelial and globet cells; (**H**) Weak immunoreactivity of the cells from the gills against the CYN. Arrows and circles indicate isolated or groups of CYN-immunopositivity cells, respectively.

After 24 h of exposure to 200 µg/Kg bw of pure CYN, the liver from those fish exposed by gavage showed fewer immunopositive cells against the cyanotoxin (Figure 3A) in comparison to those from fish exposed by i.p. injection (Figure 3E). The immunopositivity appeared mainly in the pancreatic acini (both routes) and the hepatocytes (i.p. route). After five days of exposure, CYN immunostaining appeared to be more intense in the centrilobular hepatocytes and also appeared in the erythrocytes, this staining being higher in those animals exposed by the i.p. route (Figure 4E) in comparison to the oral route (Figure 4A). The CYN-positive hepatocytes exhibited a stronger granular staining in the cytoplasm than in the nuclei, together with a cytoplasmic eosinophilic condensation aggregated along the outer nuclear membrane.

In the kidney, no presence of CYN-positive cells was detected 24 h after the exposure to the toxin by any of the intoxication routes assayed (Figure 3B,F). However, the i.p. administration of CYN gave rise to the appearance of more CYN-immunoreactive cells in comparison to the oral administration at five days post-exposition. The staining in fish exposed by the oral route was primarily localized in proximal and distal convoluted tubules (Figure 4B), whereas by the i.p. route, CYN was mainly present in glomeruli and erythrocytes (Figure 4F).

Intestine samples after 24 h and five days from fish exposed orally appeared totally clear in both cases (Figure 3C and Figure 4C), as well as the intestine from those fish exposed i.p. and euthanized after 24 h (Figure 3G). Only the samples from fish exposed by i.p. injection and euthanized after five days presented immunopositive results (Figure 4G). In this case, CYN distribution was mainly confined to the epithelium, showing a moderate presence of CYN-positive epithelial and goblet cells.

The immunohistochemical study of the gills revealed that the targeted cells for CYN were both epithelial and goblet cells, and in a lesser extent, erythrocytes from the secondary lamellae. Staining had a cytoplasmic location with a dark granular appearance homogeneously distributed. This immunolabeling was only evident in both groups of animals euthanized at 5 days post-exposition (Figure 4D,H).

#### 2.1.2. Sub-Chronic Dose Assay

In the positive control tissue samples, CYN-specific labeling appeared as evenly distributed dark red granules or as diffuse homogeneous staining distributed in the cytoplasm of the cells. The distribution of CYN, mainly associated with the organs studied, is summarized in Table 2.

**Table 2:** Distribution of immunolabeled cells for CYN in different organs from tilapia (*Oreochromis niloticus*) exposed by immersion to 10 or 100 µg/L of CYN contained in a lyophilized CYN-producer *A. ovalisporum* strain during 7 or 14 days. Results are expressed as number of immunolabeled cells per area of 0.2 mm^2^: - absent, + scarce (0–10), ++ moderate (10–50), +++ intense (>50). **C7d:** Control fish euthanized after 7 days; **10C7d:** Fish exposed to 10 µg/L of CYN from a CYN-producer *A. ovalisporum* strain and euthanized after 7 days; **100C7d:** Fish exposed to 100 µg/L of CYN from a CYN-producer *A. ovalisporum* strain and euthanized after 7 days; **C14d:** Control fish euthanized after 14 days; **10C14d:** Fish exposed to 10 µg/L of CYN from a CYN-producer *A. ovalisporum* strain and euthanized after 14 days; **100C14d:** Fish exposed to 100 µg/L of CYN from a CYN-producer *A. ovalisporum* strain and euthanized after 14 days.

	C7d	10C7d	100C7d	C14d	10C14d	100C14d
**Liver**						
Hepatocytes	-	+++	+++	-	+++	+++
Pancreatic acini	-	++	+++	-	-	+++
Erythrocytes	-	-	++	-	+	+
**Kidney**						
Tubules	-	-	+++	-	++	+++
Glomeruli	-	-	++	-	+++	+++
Erythrocytes	-	-	++	-	+	+
**Intestine**						
Epithelium	-	+	+	-	+++	+++
**Gills**						
Secondary lamellae	-	+	++	-	++	+++
Erythrocytes	-	+	+	-	-	+

**Figure 5:**
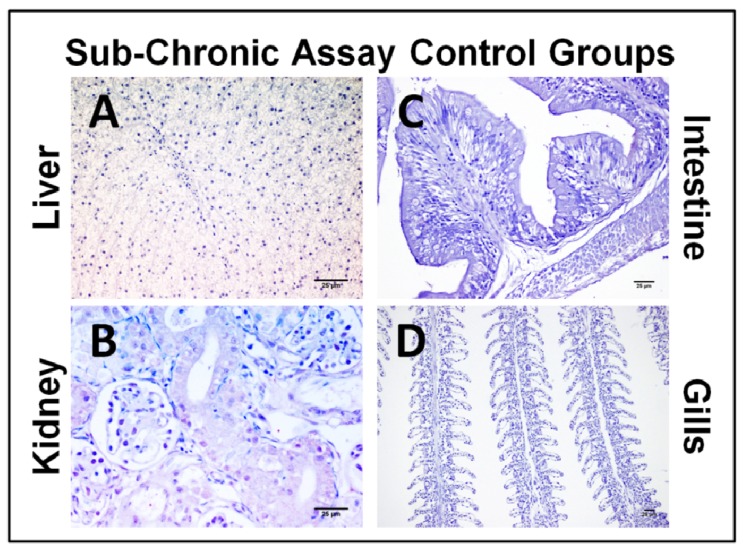
Photomicrographs of the immunohistochemistry of CYN in liver (**A**), kidney (**B**), intestine (**C**), and gills (**D**), from tilapia used as control in the sub-chronic assay. Bars: 25 µm. No labeling was observed in any of the organs studied.

There was no CYN-specific labeling in any control samples when rabbit anti-CYN serum was replaced by rabbit non-immune serum in the immunohistochemical study. CYN antigen was not detected in any of the tissue sections analyzed from fish not exposed to the toxin (Figure 5).

**Figure 6:**
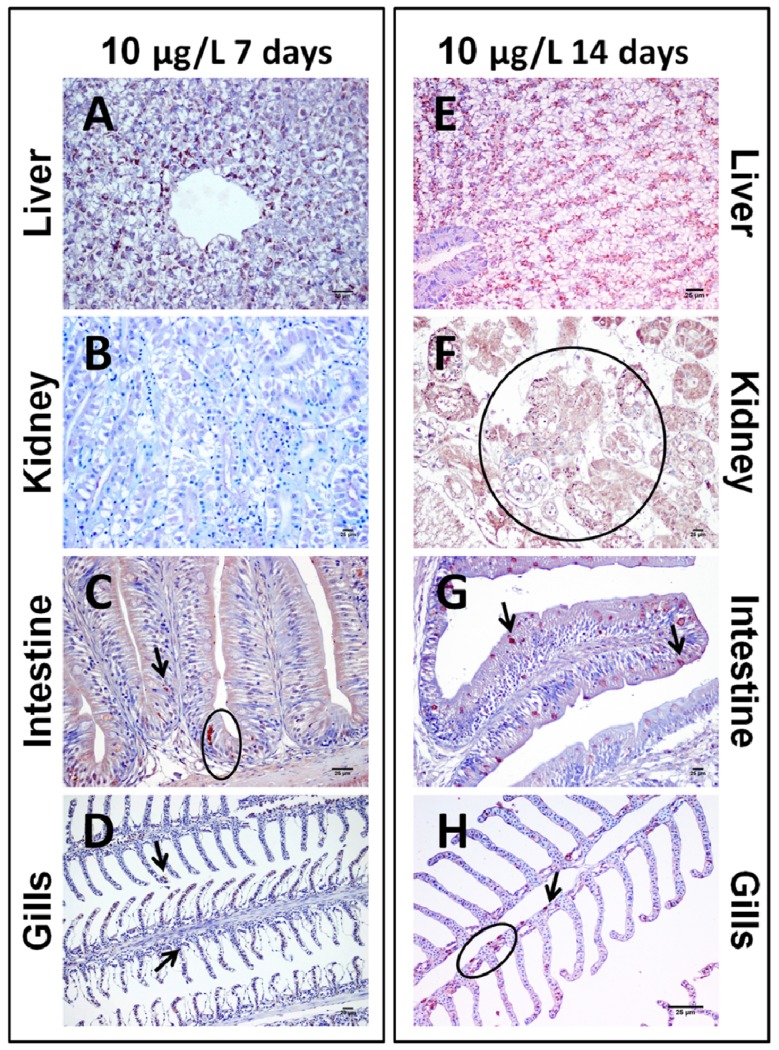
Photomicrographs of immunohistochemistry of liver (**A**,**E**), kidney (**B**,**F**), intestine (**C**,**G**), and gills (**D**,**H**) from tilapia exposed by immersion during seven days (**A**–**D**) or 14 days (**E**–**H**) to 10 µg/L of CYN contained in a lyophilized *Aphanizomenon ovalisporum* culture. Bars: 25 µm. (**A**) Moderate staining of the hepatocytes from fish exposed during seven days to CYN; (**B**) Absence of immunoreactive cells against CYN in the renal parenchyma; (**C**) Slight immunostaining in the intestine, mainly localized in the epithelial and globet cells; (**D**) Weak immunolabeling in cells from gills; (**E**) Mild CYN-positive staining of the hepatocytes from liver exposed during 14 days; (**F**) Strong immunostaining in cells from the proximal and distal convoluted tubules, and in cells from the glomeruli of the kidney; (**G**) Considerable immunostaining of the intestinal cells, mainly localized in the epithelial and globet cells; (**H**) Positive staining of cells from gills. Arrows and circles indicate isolated or groups of CYN-immunopositivity cells, respectively.

**Figure 7:**
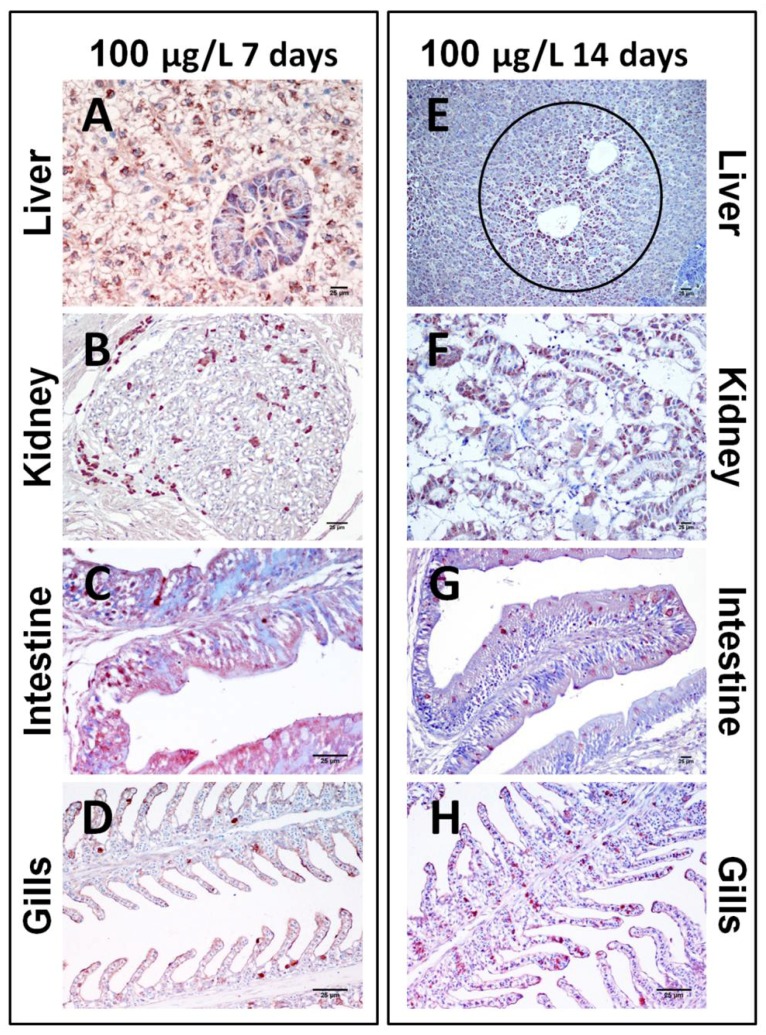
Photomicrographs of immunohistochemistry of liver (**A**,**E**), kidney (**B**,**F**), intestine (**C**,**G**), and gills (**D**,**H**) from tilapia exposed by immersion during seven days (**A**–**D**) or 14 days (**E**–**H**) to 100 µg/L of CYN contained in a lyophilized *Aphanizomenon ovalisporum* culture. Bars: 25 µm. (**A**) Intense staining of the hepatocytes from fish exposed during seven days to CYN; (**B**) Immunoreactive cells against CYN in the renal parenchyma; (**C**) Slight immunostaining in the intestine, mainly localized in the epithelial and globet cells; (**D**) Immunolabeling in cells from gills; (**E**) Strong CYN-positive staining of the hepatocytes from liver exposed during 14 days; (**F**) Intense positive immunostaining in cells from the proximal and distal convoluted tubules, and in cells from the glomeruli of the kidney; (**G**) CYN-positive cells of the intestinal lumen, mainly in the epithelial and globet cells; (**H**) Positive staining of cells from gills. Circles indicate CYN-immunopositive cells.

The immunohistochemical study of fish exposed by immersion to two different concentrations of CYN from an *A. ovalisporum* strain for 7 or 14 days revealed that the liver was the organ with most CYN-positive cells. This staining was concentration-dependent for both considered periods of time (Figure 6A,E). Thus, a slight minor quantity of CYN-positive cells was detected in the animals exposed to 10 µg CYN/L, without the influence of the exposure time. At 7 and 14 days, in fish exposed to 100 µg CYN/L the same areas were stained, and erythrocytes, pancreatic islet cells and, most frequently, hepatocytes were CYN-positives. Such cytoplasmic granular staining observed in hepatocytes was detected in almost all the lobules, these cells being enlarged and distributed especially adjacent to the central vein (Figure 7A,E).

Immunolabeling in kidney displayed differences between fish subjected to different concentrations of CYN at the same time of exposure, and between fish subjected to the same concentration of CYN at different times. In those fish exposed to 10 µg CYN/L for seven days, the toxin was not immunohistochemically detectable in tissue sections (Figure 6B). On the contrary, when fish were exposed for 14 days, a strong staining in the cytoplasm of proximal and distal convoluted tubules, as well as in the glomeruli was observed (Figure 6F). In those fish exposed to the highest concentration of CYN (100 µg/L), there was an increase in the number of CYN-positive cells at both exposure times (Figure 7B,F)

In the intestine, the presence of CYN-immunopositive cells was time-dependent. Thus, after seven days of the exposure, the intestine of fish intoxicated with both doses of CYN showed a slight quantity of epithelial and goblet cells with granular staining in the epithelium (Figure 6C and Figure 7C), while fish exposed to both concentrations for 14 days showed an increase of the CYN-specific labeling, mainly in the goblet cells (Figure 6G and Figure 7G).

Finally, the immunohistochemical study of the gills showed that the main CYN-positive cells were epithelial and goblet cells from the secondary lamellae and erythrocytes. The immunolabeling was more pronounced with the highest concentration of CYN used (100 µg/L) and the longest time of exposure (14 days) (Figure 7H).

### 2.2. Discussion

Studies concerning CYN are increasing due to its recent increase in freshwater bodies all over the world. There are several studies regarding the CYN-toxic effects in different animal models [12,23], but studies related to its distribution in organisms and the relation with the damage induced are still scarce. To our knowledge, this is the first study that investigates by an immunohistochemical technique the distribution of CYN in fish acutely (gavage and i.p. injection) or sub-chronically (immersion) exposed to pure CYN or to a CYN-producing culture, respectively.

Regarding our work, CYN-immunostaining appeared in all the organs studied (liver, kidney, intestine, gills) in both the acute and the sub-chronic assays, with the exception of the heart. In both experiments, the order of the intensity of the CYN-positive staining in all the organs was as follows: liver > kidney > intestine > gills.

The highest liver CYN-immunopositivity correlates with the histopathological lesions described by light and electron microscopy in tilapia (*O.* *niloticus*) acutely and sub-chronically exposed to the same conditions as described in this work [30,39]. These histopathological alterations were also consistent with the exposure route and the time of euthanization. The liver appeared to be the main target organ, as previously stated by Fischer *et al*. [34]. This study also indicated that the liver was the main target organ for the cyanotoxin MC-LR in rainbow trout (*Oncorrhynchus mykiss*) orally-exposed to 5.7 mg/Kg bw of MC-LR from a cyanobacterial strain. In the same way, Ito *et al*. [40] also found that liver from mice exposed by the same route to 0.5 mg/Kg bw of MC-LR was the organ presenting the most intense immunolabeling, especially the cytoplasm from the hepatocytes located around the central vein, as we also found in the experiments carried out in the present work. Yoshida *et al*. [32] correlated the immunopositivy in the liver with some injuries, such as hemorrhage and apoptosis, in mice i.p. administered with MC-LR.

Following the liver, the kidney was the organ with more CYN-immunopositive cells, corroborating the selectivity of the CYN for this organ, as other authors stated before in mice and fish [29,41]. Gutiérrez-Praena *et al*. [30] and Guzmán-Guillén *et al*. [39] also corroborated these findings through histopathology of the kidney of *O. niloticus* treated under the same conditions as in the present experiments. The distribution of CYN described in this work correlates, mostly, with the histopathological findings described. Moreover, studies of the body distribution of ^14^C-labeled CYN in mice showed that CYN was mainly excreted through the kidney, as nearly 50% of an i.p. administered dose of CYN appeared in the urine in 6 h, whereas 20% of the dose was present in the liver [42].

Concerning the intestine, we observed that staining appeared in a low range in comparison to liver and kidney, and mainly in the epithelium. This is in agreement with the histopathological results presented by Gutiérrez-Praena *et al*. [30] and Guzmán-Guillén *et al*. [39] as the localization of CYN in the epithelium correspond with the main damage induced in the intestine. Ito *et al*. [40] described a MC-LR-immunopositivity in the epithelial and globet cells from the intestine of mice. However, Lance *et al*. [35] found that the intestine of the snail *Lymnaea stagnalis* presented an intense MC-LR-immunopositive staining derived from pure MC-LR or MC-LR extracted from a toxic cyanobacteria strain. This fact could indicate a barrier function of the intestine, reducing the damage produced by the toxins in other organs. This has not been observed in either of our two studies.

When comparing both assays, some differences appeared in relation to the immunostaining of the organs. Thus, the positive staining in liver and kidney was more intense in those fish from the sub-chronic assay than in those from the acute assay, being even absent in those fish euthanized after 24 h. Although these differences could be due to the kind of CYN administered to fish (pure or producing cells), it is also probable that the exposure time plays a role in them. Thus, fish exposed for 14 days to CYN presented in general the most intense signal in both organs. The differences were also noticeable in the intestine, where a more intense staining appeared in fish from the sub-chronic assay. In this organ, these differences could also be explained by the exposure route. Hence, when CYN was i.p. administered, the intestine was not a usual absorption route, and when it comes to gavage it is probably that the unique dose of pure toxin crosses the membranes and enters into the blood stream. However, CYN from the cyanobacterial strain appeared evident from seven days of administration, which could be due to the continuous presence of the toxin in the water. This last statement could be also applied to gills, since fish from the sub-chronic assay are continuously filtrating water containing CYN, whereas fish from the acute assay only presented CYN-positive cells after a five-day exposure.

## 3. Experimental Section

### 3.1. Chemicals

Pure Cylindrospermopsin (purity ≥ 95%) was supplied by Alexis Corporation (Lausen, Switzerland). All chemicals were provided by Sigma-Aldrich (Madrid, Spain) and VWR International Eurolab (Seville, Spain).

### 3.2. *Aphanizomenon Ovalisporum* Culture and Determination of Cyanobacterial Toxins

*A. ovalisporum* (LEGE-X001) strain was supplied by the Centro Interdisciplinar de Investigação Marinha e Ambiental (CIIMAR, Porto, Portugal). A culture of this strain was maintained in Z8 medium at 25 °C under continuous illumination with an intensity of 28 µmol photons m^−2^ s^−1^ provided by cool white fluorescent tubes. After 33 days, cultures were harvested by decantation with a plankton-net (20 µm diameter). The biomass obtained was frozen at −80 °C until lyophilization (Telstar Cryodos, Madrid, Spain). 

Cylindrospermopsin was extracted from the dried cell material following the Guzmán-Guillén *et al*. [43] method. The liquid chromatography (LC) system used to analyze the toxin content was a Varian 9012 equipped with a Varian ProStar 330 Diode Array Detector (DAD, Varian Technologies, Palo Alto, CA, USA). Chromatographic data were processed with a Star Chromatography Workstation (Varian Technologies, Palo Alto, CA, USA). Chromatographic separation of CYN was performed according to Guzmán-Guillén *et al*. [43] on a 250 mm × 4.6 mm internal diameter, 5 µm, LiChrosphere C18 column purchased from Merck (Darmstadt, Germany). The standard solutions of CYN were prepared in water (100 µg/mL) and diluted as required with water for their use as working solutions (0.08–5.0 µg/mL). After analysis, the concentration of CYN obtained from the lyophilized cells was 8.7 µg/mg.

### 3.3. Experimental Setup and Acclimation of Fish

The experiment had the approval of the Ethic Committee of the University of Seville. *Oreochromis niloticus* (Nile tilapia, Cichlidae) were obtained from Valenciana de Acuicultura (Valencia, Spain). Fish were transferred to the laboratory, where they were held in glass aquaria, with 96 L of dechlorinated tap water, a continuous system of water filtration and aeration (Eheim Liberty 150 Bio-Espumador cartridges (Eheim, Stuttgart, Germany), and a 12:12-h light:dark photoperiod. Temperature was maintained at 21 ± 2 °C and dissolved oxygen at 7.0 ± 0.5 mg/L. Mean values for additional water-quality parameters were pH 7.6 ± 0.3, conductivity of 287 µS/cm, 0.60 mM Ca^2+^, and 0.3 mM Mg^2+^. Fish were fed with commercial fish food (Dibaq, Segovia, Spain) containing 6% lipids, 31% proteins, 37% carbohydrates, 2.5% fiber, 1.5% total phosphorus, 12% ash, 200 mg α-tocopherol/kg, 1700 IU vitamin D3/kg feed, and 10,000 IU vitamin A/kg feed. Fish were acclimatized for 15 days before the beginning of the experiments. For the acute assay sixty-four fish (53.4 ± 5.2 g; length: 12 ± 3 cm) were used, whereas forty-eight fish were needed in for the sub-chronic experiment (50 ± 8 g; length: 12 ± 2 cm). In both cases, fish were randomly distributed in aquaria (*n* = 8 individuals/aquarium).

### 3.4. Experimental Exposure

For the acute assay (Table 3), fish in aquaria 3 and 7 were exposed by gavage to a single dose of 200 µg/Kg of pure CYN in 0.5 mL of 0.9% (*w*/*v*) NaCl solution and were euthanized after 24 h (aquarium 3) and five days (aquarium 7). Control fish were allocated to aquaria 1 and 5, received only the vehicle solution (0.5 mL of 0.9%, *w*/*v*, NaCl), and were euthanized after 24 h (aquarium 1) and five days (aquarium 5). Similarly, two other groups of fish were exposed by i.p. injection to 200 µg/Kg bw of pure CYN in 0.5 mL of 0.9% (*w*/*v*) NaCl solution and were euthanized after 24 h (aquarium 4) and five days (aquarium 8). The corresponding control groups were injected with the vehicle only and were euthanized after 24 h (aquarium 2) and five days (aquarium 6).

**Table 3:** Feeding conditions of *Oreochromis niloticus* and exposure pathways to pure Cylindrospermopsin (CYN). Numbers from 1 to 8 correspond with the aquaria where the different treatments were carried out. (+ With; - Without).

	24 h				5 days			
	1	2	3	4	5	6	7	8
NaCl solution by gavage	+	-	-	-	+	-	-	-
NaCl solution by i.p. injection	-	+	-	-	-	+	-	-
Pure CYN by gavage	-	-	+	-	-	-	+	-
Pure CYN by i.p. injection	-	-	-	+	-	-	-	+

For the sub-chronic assay (Table 4), fish in aquaria 2 and 5 were fed with commercial fish food and exposed by immersion to an adequate quantity of cyanobacterial cells at the beginning of the experiment in order to obtain 10 µg CYN/L. Fish in aquaria 3 and 6 were fed similarly but with enough cyanobacterial cells to obtain 100 µg CYN/L. Fish from aquaria 1 and 4 were fed only with the commercial food. After 7 days (aquaria 1, 2, and 3) and 14 days (aquaria 4, 5, and 6), fish were euthanized.

**Table 4:** Feeding conditions of *Oreochromis niloticus* and exposure to Cylindrospermopsin (CYN) contained in a lyophilized *Aphanizomenon ovalisporum* strain. Numbers from 1 to 6 correspond with the aquaria where the different treatments were carried out. (+ With; - Without).

	7 days			14 days		
	1	2	3	4	5	6
10 µg/L of CYN	-	+	-	-	+	-
100 µg/L of CYN	-	-	+	-	-	+

### 3.5. Immunization with Cylindrospermopsin (CYN) and Preparation of Polyclonal Antiserum

Polyclonal antiserum was prepared according to the method of Hancock and O’Reilly [44]. Rabbit females (*Orhyctolagus cuniculus*, breed New Zealand White) weighing 1.8 to 2.0 kg were maintained in sterile conditions a week before the start-up of the immunization protocol to become accustomed. Then, they received two intravenous administrations into the marginal ear vein with seven days apart of a solution of 100 µg of pure CYN in 0.5 mL of phosphate buffered saline solution **(**PBS). Blood samples were taken from the central ear artery at 14 days post-inoculation, allowed to clot at room temperature for 1 h and then centrifuged at 3000 rpm for 10 min to obtain the serum, which was stored at −20 °C until assayed.

### 3.6. Indirect ELISA

In order to elucidate the specificity of the anti-CYN antibody, an indirect ELISA was carried out. First of all, since CYN is not a good immunogen, it was coupled to keyhole limpet hemocyanin (KLH) from *Megathura crenulata*, following the procedure described by Elliot *et al*. [45]. After that, the same competitive indirect ELISA protocol followed by these authors was performed. Different dilutions of the positive serum (containing anti-CYN antibody) and the negative serum (without anti-CYN antibody) were screened in order to study the sensitivity of the anti-CYN antibody. The dilutions assayed were 0, 1/10, 1/20, 1/50, 1/100, 1/200, 1/500, and 1/1000. The study demonstrated the specificity of the anti-CYN antibody of the serum for the CYN, as well as a good sensitivity at the dilution 1/100 (data not shown).

### 3.7. Immunohistochemical Analyses for the Detection of CYN in Tissues

For the immunohistochemical examinations, tissue samples (0.5–1 cm thick) were taken from the organs of control and exposed fish. These samples were fixed in 10% neutral buffered formalin for 24 h at 4 °C, and then they were immediately dehydrated in a graded series of ethanol, immersed in xylol, and embedded in paraffin wax using an automatic processor. The immunohistochemical studies were always performed by the same person using the avidin-biotin-peroxidase complex (ABC) method, where the samples of each organ were analyzed the same day and with the same reagents. Briefly, tissue sections (3 μm) were dewaxed and rehydrated through a graded ethanol descendent series. Although endogenous peroxidase activity is almost completely destroyed during formalin fixation, this was exhausted by incubation of the samples with H_2_O_2_ 3% in methanol for 45 min at room temperature (RT) to abolish pseudoperoxidase activity of red blood cells and peroxidase activity in myeloid cells, avoiding as well the presence of background in order to facilitate the interpretation of the immunologic reaction [37,46]. To achieve satisfactory results, tissue sections were subjected to different unmasking methods for retrieving the antigen masked by the formalin fixation and increasing the permeability of tissues to the anti-toxin serum, being the most effective the 0.01 M citrate buffer (pH = 6) for 30 min at 37 °C in oven (Table 5). Then, samples were submitted to three rinses of 10 min each one in PBS and covered with 30% normal goat serum (ICN Biomedicals, Aurora, OH, USA) in PBS for 30 min at RT to avoid background. Tissue detection of CYN was carried out by incubation of the samples with different rabbit anti-CYN serum dilutions in PBS at 4 °C overnight, obtaining the best results with the 1:100 serum dilution (Table 5).

**Table 5:** Immunoreactivity of the serum containing anti-CYN in tilapia tissues. The different pre-treatments used were: (**a**) sections not subjected to antigen retrieval; (**b**) Tween 20^®^ (Merck, München, Germany) 0.1% in 0.01M PBS, pH 7.2, for 10 min at RT; (**c**) protease type XIV (Sigma-Aldrich Chemie, Steinheim, Germany) 0.1%, pH 7.2, for seven min at RT; incubation with 0.1 M citrate buffer (CB) in oven for 30 min at 37 °C; (**d**) microwave heating in 0.01 M CB, pH 3.2, 6 or 9 (6 or 15 min from the beginning of boiling). **^†^** (-) no positivity; (+) light positivity and light background; (++) positive reaction and light background; (+++) positive reaction without background; (Bs) positive reaction but intense background of the staining.

Antigen retrieval method		Dilutions of anti-CYN rabbit serum ^†^			
		1:10	1:50	1:100	1:200
**None**		-	-	-	-
**Tween 20**^®^		-	-	-	-
**Protease**		-	-	-	-
**CB pH 3.2**	oven	+	+	+	+
	microwave (6 min)	+	+	+	-
	microwave (15 min)	-	-	-	-
**CB pH 6**	oven	Bs	Bs	+++	+
	microwave (6 min)	Bs	++	++	+
	microwave (15 min)	+	+	+	-
**CB pH 9**	oven	-	-	-	-
	microwave (6 min)	-	-	-	-
	microwave (15 min)	-	-	-	-

After primary incubation, slides were washed in PBS (three times for 10 min each) and incubated with the biotinylated goat anti-rabbit IgG secondary antibody (Vector Laboratories, Burlingame, CA, USA) diluted 1:200 in PBS for 30 min at RT. After three further washes in PBS, samples were incubated with the avidin-biotin-peroxidase complex (Vectastain^®^ ABC Kit Elites, Vector Laboratories, Peterborough, United Kingdom) for 1 h at RT in the dark. All tissue sections were finally rinsed in 0.05 M Tris buffered saline (TBS), pH 7.6, and labeling was visualized by application of a chromogen solution (NovaRED^®^ Substrate Kit, Vector Laboratories, Peterborough, United Kingdom). Slides were counterstained with Mayer’s haematoxylin.

As a negative control of the primary antibody, this was replaced in positive tissue sections by rabbit non-immune serum (DakoCytomation, Glostrup, Denmark) or by PBS followed by incubation with secondary antibodies and detection reagents. Moreover, tissue negative controls were included using tissue samples from different organs of fish not submitted to CYN exposure. Positive control tissues were from both control fish used on each experiment.

The identification of target cells for the toxin was based on morphological features, location, and size of the cells. Assessment of the immunolabeled cells was performed in 25 fields of 0.2 mm^2^ randomly chosen, being the results expressed as number of immunolabeled cells per area of 0.2 mm^2^, as well as: - (absent), + or scarce (0–10), ++ or moderate (10–50), +++ or intense (>50). These quantifications were performed by two experienced observers but with no previous knowledge of which group was being analyzed.

## 4. Conclusions

The present immunohistochemical study demonstrates a similar pattern of CYN distribution in both experiments, presenting most immunopositive results in the liver, followed by the kidney, intestines, and gills. Moreover, it suggests that fish organs subjected to a sub-chronic exposure of CYN, presented a stronger staining in comparison to the acute exposure. In general, immunolabeling intensified with increasing time in the acute assay, confirming the delayed toxicity of CYN, and also with the increment of the dose, as it is shown in the sub-chronic assay, where the staining was more pronounced with the highest concentration of CYN assayed (100 µg/L) and the longest time of exposure (14 days). Noteworthy are the differences in the immunolabeling in the intestine and gills between both assays, with more CYN-immunopositive cells appearing in fish from the sub-chronic assay, which is explained by the exposure route, as the fish are constantly immersed in the water containing the toxin. Finally, we also conclude that immunohistochemistry may be a useful tool for detecting and monitoring CYN in tissues, helping to establish the target organs for this toxin.
